# Dopamine/BDNF loss underscores narcosis cognitive impairment in divers: a proof of concept in a dry condition

**DOI:** 10.1007/s00421-022-05055-6

**Published:** 2022-10-10

**Authors:** Gerardo Bosco, Tommaso Antonio Giacon, Nazareno Paolocci, Alessandra Vezzoli, Cinzia Della Noce, Matteo Paganini, Jacopo Agrimi, Giacomo Garetto, Danilo Cialoni, Natalie D’Alessandro, Enrico M. Camporesi, Simona Mrakic-Sposta

**Affiliations:** 1 Department of Biomedical Sciences, University of Padova, 35131 Padova, Italy; 2 Division of Cardiology, Johns Hopkins Medical Institutions, Baltimore, MD USA; 3 Institute of Clinical Physiology, National Research Council (CNR), 20162 Milano, Italy; 4 ATIP Center for Hyperbaric Medicine, Padova, Italy; 5 Dan Europe Foundation, Research Division, Roseto degli Abbruzzi, Teramo, Italy; 6 Team Health, TGH, Tampa, FL USA

**Keywords:** Narcosis, Deep diving, Brain-derived neurotrophic factor (BDNF), Dopamine, Reactive oxygen species (ROS)

## Abstract

**Purpose:**

Divers can experience cognitive impairment due to inert gas narcosis (IGN) at depth. Brain-derived neurotrophic factor (BDNF) rules neuronal connectivity/metabolism to maintain cognitive function and protect tissues against oxidative stress (OxS). Dopamine and glutamate enhance BDNF bioavailability. Thus, we hypothesized that lower circulating BDNF levels (via lessened dopamine and/or glutamate release) underpin IGN in divers, while testing if BDNF loss is associated with increased OxS.

**Methods:**

To mimic IGN, we administered a deep narcosis test via a dry dive test (DDT) at 48 msw in a multiplace hyperbaric chamber to six well-trained divers. We collected: (1) saliva samples before DDT (T0), 25 msw (descending, T1), 48 msw (depth, T2), 25 msw (ascending, T3), 10 min after decompression (T4) to dopamine and/or reactive oxygen species (ROS) levels; (2) blood and urine samples at T0 and T4 for OxS too. We administered cognitive tests at T0, T2, and re-evaluated the divers at T4.

**Results:**

At 48 msw, all subjects experienced IGN, as revealed by the cognitive test failure. Dopamine and total antioxidant capacity (TAC) reached a nadir at T2 when ROS emission was maximal. At decompression (T4), a marked drop of BDNF/glutamate content was evidenced, coinciding with a persisting decline in dopamine and cognitive capacity.

**Conclusions:**

Divers encounter IGN at – 48 msw, exhibiting a marked loss in circulating dopamine levels, likely accounting for BDNF-dependent impairment of mental capacity and heightened OxS. The decline in dopamine and BDNF appears to persist at decompression; thus, boosting dopamine/BDNF signaling via pharmacological or other intervention types might attenuate IGN in deep dives.

## Introduction

Diving at great depths (or the permanence at high pressures) induces some peculiar perturbations in the cognitive domain. This evidence dates back to 1935 when Behnke and colleagues first associated this phenomenon with increased inert gas accumulation, mainly N_2_ (Grover et al. 2014). Now, we know that pressures greater than 4 absolute atmospheres (ATA) (30 m of seawater (msw) = partial N_2_ pressure of ≅ 3.1 ATA) can usher in inert gas narcosis (nitrogen narcosis, IGN). IGN is a reversible change in consciousness that occurs while diving at deep depths. Certain gases at high pressure cause an anesthetic effect that alters a diver's consciousness (Kirkland et al. 2021; Moon et al. 2009; Vann et al. 2011).

IGN encompasses subjective and objective mental disturbance (Clark 2015) and can manifest during ﻿self-contained underwater breathing apparatus (SCUBA) diving. Since divers inhale gas mixtures at different pressures, in theory, and practice, IGN could be blunted by using N_2_-deprived gas mixtures, such as helium–oxygen (Bennett et al. 1975; Gelfand et al. 1983; Kirkland et al. 2021). However, elite breath-hold divers can reach extreme depths nowadays, and they also report IGN symptoms when trying to stretch their limits in record-breaking dives (Freiberger et al. 2016; Lindholm et al. 2009). Hence, factors accounting for IGN genesis remain worth investigating.

IGN can impair cognitive functions and physical performance from depths as low as 10 m, becoming more apparent at 30–40 m depth (Rostain et al. 2011), and it remains one of the leading causes of underwater accidents (Unsworth 1966; Ranapurwala et al. 2014; Clark 2015). Importantly, IGN occurs especially during recreational diving at depths greater than 40 msw (Vann et al. 2011). Clinically, it includes several psychomotor disorders, with reversible alterations (Turle et al. 1998). Its symptoms closely resemble the cognitive, neuromuscular, and vestibular alterations experienced by subjects drinking alcohol, being mild and benign at shallow depths and more severe and debilitating when divers go deeper (Clark 2015). The Meyer–Overton hypothesis is at the roots of IGN neurochemistry; this hypothesis is based on the correlation between anesthetic potency and lipid solubility, and the anesthetic potency is proportional to the lipid/water partition coefficient (Meyer 1899; Overton & Lipnick 1991). Furthermore, the lipid solubility of inert gases is one of its key postulations (Bennett et al. 1989; Rostain et al. 2011). However, this rule suffers from several weaknesses; in fact, stereoisomers of an anesthetic can have very different narcotic potency, even though their oil/gas partition coefficients are similar (Cameron 2006).

Over the years, IGN pathogenesis has been linked to alterations in GABAa and glutamatergic NMDA neuronal receptors systems (Rostain et al. 2016). For example, changes in the extracellular concentrations of GABA have also been described during exposure to hyperbaric oxygen (HBO_2_) at 5 ATA (Zhang et al. 2004). Similarly, declines in norepinephrine (NE) and dopamine (DA) release occur in the rat caudate nucleus and hypothalamus after exposure to a nitrogen–oxygen mixture at 20 ATA (McLeod et al. 1988). Nevertheless, all this evidence always comes from the rat and/or scuba and technical divers (Yang et al. 2002; Rocco et al. 2019).

Ambient pressure increases by one ATA at depth every 10 msw; therefore, a hyperbaric chamber approach can conveniently circumvent these hurdles. Indeed, it allows higher concentrations of gases to be inhaled and dissolved into cells, membranes, and body fluids under dry conditions, de facto mimicking what occurs in deep divers in the sea.

Brain-derived neurotrophic factor (BDNF) is essential for neuronal growth, differentiation, and connectivity, acting as an on-demand tissue protectant under stress conditions (Agrimi et al. 2019; Kowianski et al. 2018; Rothman et al. 2013). It—directly or indirectly (via glutamate excitatory neurotransmission)—preserves cognitive function (Benarroch et al. 2015; Miranda et al. 2019). BDNF is also vital in maintaining normal cardiac contraction and relaxation (Agrimi et al. 2019; Feng et al. 2015) and shields tissues against oxidative stress. For instance, in schizophrenic patients, a marked decline in circulating BDNF levels correlates with low levels of antioxidants, such as superoxide dismutase and glutathione peroxidase, and with a substantial rise in malondialdehyde (MDA) and end products of lipid peroxidation (Zhang et al. 2015). Finally, many neurotransmitters, including dopamine and glutamate can enhance BDNF bioavailability in neurons (Bagayogo et al. 2009; Kuppers et al. 2001). On these grounds, we enrolled healthy (highly experienced) divers and subjected them to a simulated dive to 48 msw (5.8 ATA) inside a hyperbaric chamber to test the following three new hypotheses. First, IGN narcosis stems, at least in part, from the lack of circulating BDNF. Second, this loss is attributable to a lack of BDNF-releasing neurotransmitters, such as dopamine and glutamate; third, impoverished levels of systemic and likely local pools of BDNF contribute to heightening oxidative stress in deep divers. Ultimately, all events would drive cognitive impairment in divers at depth.

## Materials and methods

We performed the current observational study employing a dry dive test at 5.8 ATA—a depth narcosis induction test—at the ATIP hyperbaric facility in a hyperbaric multiplace chamber VECOM, Padova (Italy).

### Selections of participants

We enrolled in the protocol six well-trained scuba and breath-hold divers, with more than 5 years of high-level experience, (four males, two females): age (43.5 ± 14.6 yrs), height (174.5 ± 6.7 cm), weight (68.5 ± 12.3 kg) e BMI (22.4 ± 3.1 kg.m^−2^) (mean ± SD). Exclusion criteria were: pregnancy, smoking, alcohol, drugs, vitamin/minerals/herbs/antioxidant supplements, exercise/training in the pre-test week, and/or a SCUBA dive made within 2 weeks before the experiment.

### Ethical considerations

The subjects were recruited voluntarily (no incentives were provided to the participants) and they signed a written informed consent form. The experimental protocol was approved by the Ethical Committee of the University of Milan (Aut. #37/17) and adhered to the principles of the Declaration of Helsinki.

### Experimental protocol

**Fig. 1:**
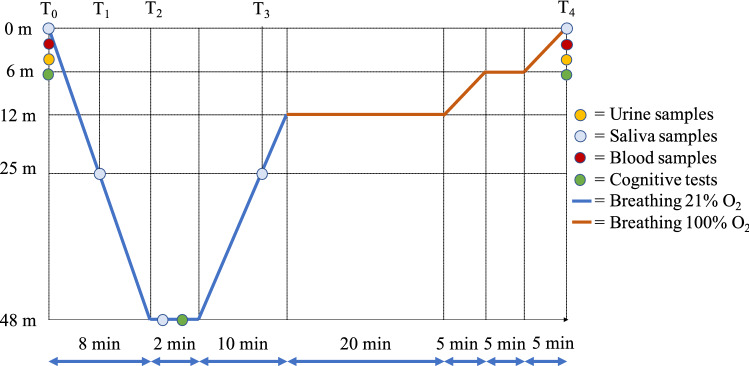
Experimental study design

On the day of the experiment, the included subjects underwent a general medical screening, anthropometric measurements, a first set of biological samplings (blood, urine, and saliva) and cognitive tests to assess their basal status (T0). Then, the participants were compressed in a hyperbaric chamber to a depth of − 48 msw (157.48 feet), breathing room air. An anesthesiologist accompanied the subjects inside the chamber to assist in case of medical issues. Saliva samples were also obtained inside the chamber at 25 msw descending (T1), at the bottom, 48 msw (T2), and 25 msw ascending (T3). Once at depth, subjects performed cognitive tests. Then, they were progressively decompressed to the surface in 45 min (Fig.  1), breathing oxygen from 12 m to the surface. Ten minutes after the end of the compression, another set of samples (blood, urine, and saliva) and cognitive tests were obtained from each subject (T4).

### Cognitive tests

To determine whether the enrolled divers can experience IGN when at depth (48 msw) in the hyperbaric chamber, thus to validate our methodological application, we administered two psycho-aptitude tests on functional domains as per visuospatial abilities and executive functions (Hegarthy et al. 2005): the intuitive geometric constructions and the labyrinth (Miyake et al. 2000, 2001; Predovan et al. 2021). These tests were administered to the diver volunteers before (T0), at the bottom of the dive (T2), and at the time of surfacing (T4). Spatial ability is the capacity to understand and remember the spatial relations among objects. In a spatial ability test (also called spatial reasoning test), the tested person is required to draw two-dimensional and three-dimensional figures by connecting the dots (Miyake et al. 2000, set of paper-and-pencil tests of spatial abilities that load on three correlated, but distinguishable factors: spatial visualization, spatial relations, and perceptual speed). Executive functions are required when the behavior or information processing is not automated and requires some control. This control may be necessary because of the unpredictability of the situation (Predovan et al. 2021). Labyrinth testing is structured as a nonverbal intellectual level test. The test consists of a series of mazes that the subject has to solve. The labyrinths are of varying complexity. The test lasts 15–60 s, allowing the subject to solve as many mazes as possible.

The participants were provided with different tests each time, so there was no possibility of 'learning' or 'condition' or 'difficulty' level impacting test performance.

### Blood, urine, and saliva sample collection

Approximately, 9 ml of venous blood was drawn from an antecubital vein, with subjects sitting or lying on a bed. Each time, samples to assess oxidative stress were collected in lithium-heparinized and EDTA tubes (Vacutainer, Becton Dickinson, USA). Plasma was separated by centrifuge (5702R, Eppendorf, Germany) at 3500*g* × 10 min at 4 °C. Samples of plasma and erythrocytes were then stored in multiple aliquots at − 80 °C until assayed. Samples were thawed only once before analysis, performed within 1 month from the collection. Urine was collected by voluntary voiding in a sterile container and stored in multiple aliquots at − 20 °C until assayed and thawed only before analysis. Blood and urine samples were collected at pre- and post-hyperbaric chambers. Approximately, 1 mL of saliva was obtained by Salivette devices (Sarstedt, Nümbrecht, Germany). The subjects were instructed on the correct use (i.e., no eating, drinking, or oral intake of drugs 1 h before sample collection) (Mrakic-Sposta et al. 2020). Saliva samples were collected before DDT (T0), at 25 msw (descending, T1), 48 msw (depth, T2), and 25 msw (ascending, T3), and 10 min after decompression (T4). Salivettes were centrifuged at 1500*g* × 20 min at 4 °C and retrieved saliva was transferred, and aliquoted, then stored at − 80 °C until assayed and thawed only once before analysis.

### Assessment of circulating BDNF, glutamate, and dopamine levels

BDNF was detected using the ELISA method according to the manufacturer's instructions (Human BDNF ELISA kit, Abcam, USA). Tertiary antibodies were conjugated to horseradish peroxidase. Wells were developed with tetramethylbenzidine and measured at 450. BDNF content was quantified against a standard curve calibrated with known amounts of protein. The detection limits were 2.4 pg.mL^−1^. Measurements were performed in duplicate and are expressed as ng.mL^−1^. According to the manufacturer's instructions, glutamate concentrations were measured using an assay kit (Glutamate ELISA KIT, LDN, Germany). Detection limits were 0.3 μg.mL^−1^. The amount of glutamate was quantified by colorimetric spectrophotometry at 450 nm against a standard curve. Measurements were performed in duplicate and are expressed as μM.L^−1^ according to the manufacturer's conversion suggested. Dopamine in plasma and saliva was determined by a kit (cat. no. EU0392; FineTest, Wuhan, China) based on a competitive ELISA detection method, sensitivity 0.938 ng.mL^−1^. Analysis was carried out according to the manufacturer's instructions. Dopamine concentration was determined using a standard curve. Samples and standards were read spectrophotometrically at a wavelength of 450 nm and concentrations are expressed in ng.mL^−1^.

### Oxidative stress

1. Reactive oxygen species (ROS) and antioxidant capacity (TAC) by electron paramagnetic resonance (EPR) in plasma and saliva
  We used electron paramagnetic resonance (EPR) spectroscopy X-band (E-Scan-Bruker BioSpin, GmbH, MA USA) to assess total ROS production and total antioxidant capacity (TAC) before and after the dry dive session (from blood samples), as well as their kinetics during timeline: at baseline (T0), descending at 25 msw (T1), depth 48 (T2), in ascending, at 25 msw (T3) and at the end (T4), using the saliva of the enrolled highly trained divers, according to previously described methods (Mrakic-Sposta et al. 2014; 2019; 2020; 2022; Bosco et al, 2018a; 2021). CMH (1-hydroxy-3-methoxycarbonyl-2,2,5,5-tetramethylpyrrolidine) spin trap probe was used for ROS determination in both fluids. A stable radical CP· (3-carboxy2,2,5,5-tetramethyl-1-pyrrolidinyloxy) was used as an external reference to convert ROS determinations into absolute quantitative values (μmol.min^−1^). TAC was measured using 1,1-diphenyl-2-picrylhydrazyl (DPPH•) quenching, in both fluids (plasma and saliva) (Mrakic-Sposta et al. 2019; 2020; Giacon et al. 2021). The calculated antioxidant capacity was expressed in terms of Trolox equivalent antioxidant capacity (TAC, mM). Samples were stabilized at 37 °C with "Bio III" unit, interfaced with the spectrometer.
2. 8-isoprostane in urine

As a marker of oxidative stress in the urine, we measured 8-isoprostane concentration (8-iso-PGF2α) by an immunoassay kit (Cayman Chemical, Ann Arbor, MI, USA) as previously described (Mrakic-Sposta et al. 2014; Bosco et al. 2019; 2021). Samples were read spectrophotometrically at a wavelength of 512 nm.

### Nitric oxide pathway

Nitrite and nitrate (NO^−2^, NO^−3^) are the end products of NO^.^ oxidation pathway. NO^.^ metabolites (NO_2_, + NO_3_ = NOx) were determined in urine via a colorimetric method based on the Griess reaction, using a commercial kit (Cayman Chemical, Ann Arbor, MI, USA) described (Green et al. 1982; Mrakic-Sposta et al. 2019; Niu et al. 2012). Samples were read at 545 nm and the concentration was assessed by a standard curve. Inducible nitric oxide synthase (iNOS) expression in plasma was determined using a human NOS_2_/iNOS ELISA kit (cat. no. EH0556; FineTest, Wuhan, China). This assay was based on sandwich enzyme-linked immune-sorbent assay technology. NOS_2_/iNOS protein synthesis was determined using a standard curve. Samples and standards were read at a wavelength of 450 nm, and the analysis was carried out according to the manufacturer's instructions.

All samples were read in duplicate and the immune-enzymatic determinations were performed by a spectrophotometer microplate reader (Infinite M200, Tecan Group Ltd., Männedorf, Switzerland).

### Thiols

Total (tot) aminothiols (Cys: cysteine; CysGly: cysteinyl glycine; Hcy: homocysteine; and GSH: glutathione) were measured in red blood cells according to previously validated methods (Dellanoce et al. 2014; Vezzoli et al. 2016). Briefly, thiol separation was performed at room temperature by isocratic HPLC analysis on a Discovery C-18 column (250 × 4.6 mm I.D, Supelco, Sigma-Aldrich, St. Louis, MOS, USA), eluted with a solution of 0.1 M acetate buffer, pH 4.0: methanol, 81:19 (v/v), at a flow rate of 1 mL.min^−1^. Fluorescence intensities were measured with an excitation wavelength at 390 nm and an emission wavelength at 510 nm, using a fluorescence spectrophotometer (Jasco, Japan). A standard calibration curve was used.

### Creatinine, neopterin, and uric acid concentration

Creatinine and neopterin concentrations were measured using high-pressure liquid chromatography (HPLC), as described (Mrakic-Sposta et al. 2019; Vezzoli et al. 2016). A Varian instrument (pump 240, autosampler ProStar 410) determined uric acid levels coupled to a UV–VIS detector (Shimadzu SPD 10-AV, λ = 240 nm) after centrifugation at 13,000 rpm for 5 min at 4 °C. Analytic separations were performed at 50 °C on a 5 µm Discovery C18 analytical column (250 × 4.6 mm I.D., Supelco, Sigma-Aldrich) at a 0.9 mL.min^−1^ flow rate. The calibration curves were linear over the range of 0.125–1 μmol.L^−1^, 0.625–20 mmol.L^−1^, and 1.25–10 mmol.L^−1^ for neopterin, uric acid, and creatinine levels, respectively. Inter-assay and intra-assay coefficients of variation were < 5%.

### Data collection and analysis

Data were analyzed using the GraphPad Prism package (GraphPad Prism 9.3.0, GraphPad software Inc., San Diego, CA) and SPSS Statistics (SPSS version 25, IBM). Kolmogorov–Smirnov testing was utilized to determine the distribution of each data set. Data were analyzed with a non-parametric test; Wilcoxon matched-pair test was used to compare the values of the assessed biomarkers at the pre- (T0) versus post-hyperbaric chamber (T4). Kinetics (from T0 to T4) by saliva samples were compared by ANOVA repeated measures, followed by Tukey's multiple comparison test to further check the groups' significance; also cognitive tests were analyzed by ANOVA and post hoc test. Spearman product–moment correlation coefficient (r) with 95% confidence intervals (CI) was used to show up possible relationships between selected parameters. Finally, the phi coefficient (ϕ) was used to evaluate the association. All data are presented as mean ± SD, and significance was determined at *p* < 0.05.

## Results

### Reaching -48msw in the hyperbaric chamber triggers IGN-like effects in healthy divers

**Fig. 2:**
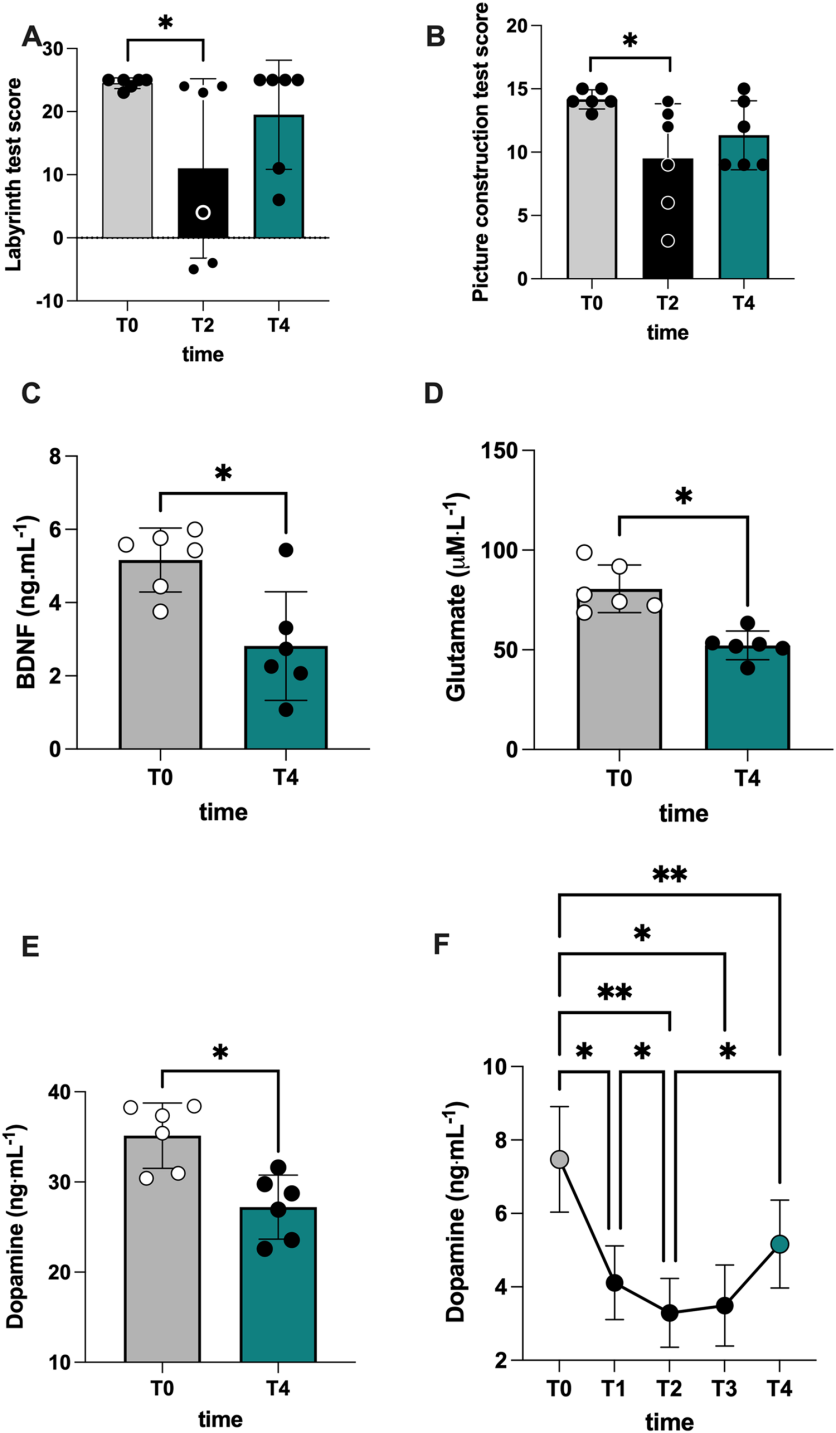
Bar charts (mean ± SD) of **A** labyrinth test score), **B** picture construction test score, at T0, T2, and T4; **C** BDNF, **D** glutamate, and **E** dopamine concentration in plasma collected at T0 and T4. Time course of **F** dopamine concentration in saliva at T0, T1, T2, T3, and T4. *p < 0.05, **p < 0.01 significantly different

In scuba divers, IGN can impair several cognitive domains (Binder et al. 2004; Martin et al. 2011; Rocco et al. 2019; Karakaya et al. 2021). Yet, what specific executive functions and neurotransmitter levels are eventually altered during and after narcosis in-depth remain to be fully deciphered. To fill this gap, we subjected the enrolled highly trained divers to a dry hyperbaric chamber diving approach to mimic a real-life "wet" IGN scenario, administering the labyrinth and Porteous tests to evaluate their cognitive functions (Miyake et al. 2000; Predovan et al. 2021). As shown in Fig. 2A, B, nitrogen narcosis at 48 msw (500 kPa) pressure in the dry hyperbaric chamber had a mild-to-moderate negative effect on divers' cognitive performance. In particular, we recorded a significant decrease (*p* < 0.05) in labyrinth test score Fig. 2A and a similar (with an increase 20% at T4 and T2) tendency in the picture construction test score Fig. 2B.

### BDNF, dopamine, and glutamate levels are reduced during a dry dive test in healthy divers

BDNF is essential for the survival and differentiation of neurons in the peripheral and central nervous systems (CNS) (Binder et al. 2004; Kowianski et al. 2018). We found that BDNF, glutamate, and dopamine plasma levels are markedly decreased in healthy highly trained divers subjected to a dry dive test at 48 msw. BDNF declined from 5.16 ± 0.87 (T0) to 2.81 ± 1.48 ng.mL^−1^ (T4), while glutamate dropped from 80.59 ± 11.97 to 52.22 ± 7.17 μM.L^−1^ and dopamine from 35.14 ± 3.61 to 27.20 ± 3.55 ng.mL^−1^ (all *p* < 0.05, Fig. 2C, D and E). Furthermore, significant differences in the saliva levels of dopamine were also found. As depicted in Fig. 2F, dopamine levels were peaking at T0 (7.47 ± 1.43 ng.mL^−1^) and plummeted over the sampling period. In detail, dopamine decreased, reaching the lowest peak value at depth (T2: 3.29 ± 0.93 ng.mL^−1^) and remaining low at T3 and T4 (T3: 3.49 ± 1.10; T4: 5.16 ± 1.19 ng.mL^−1^).

### A dry dive test triggers ROS production, while depleting antioxidant capacity in healthy divers’ saliva and blood, respectively

**Fig. 3:**
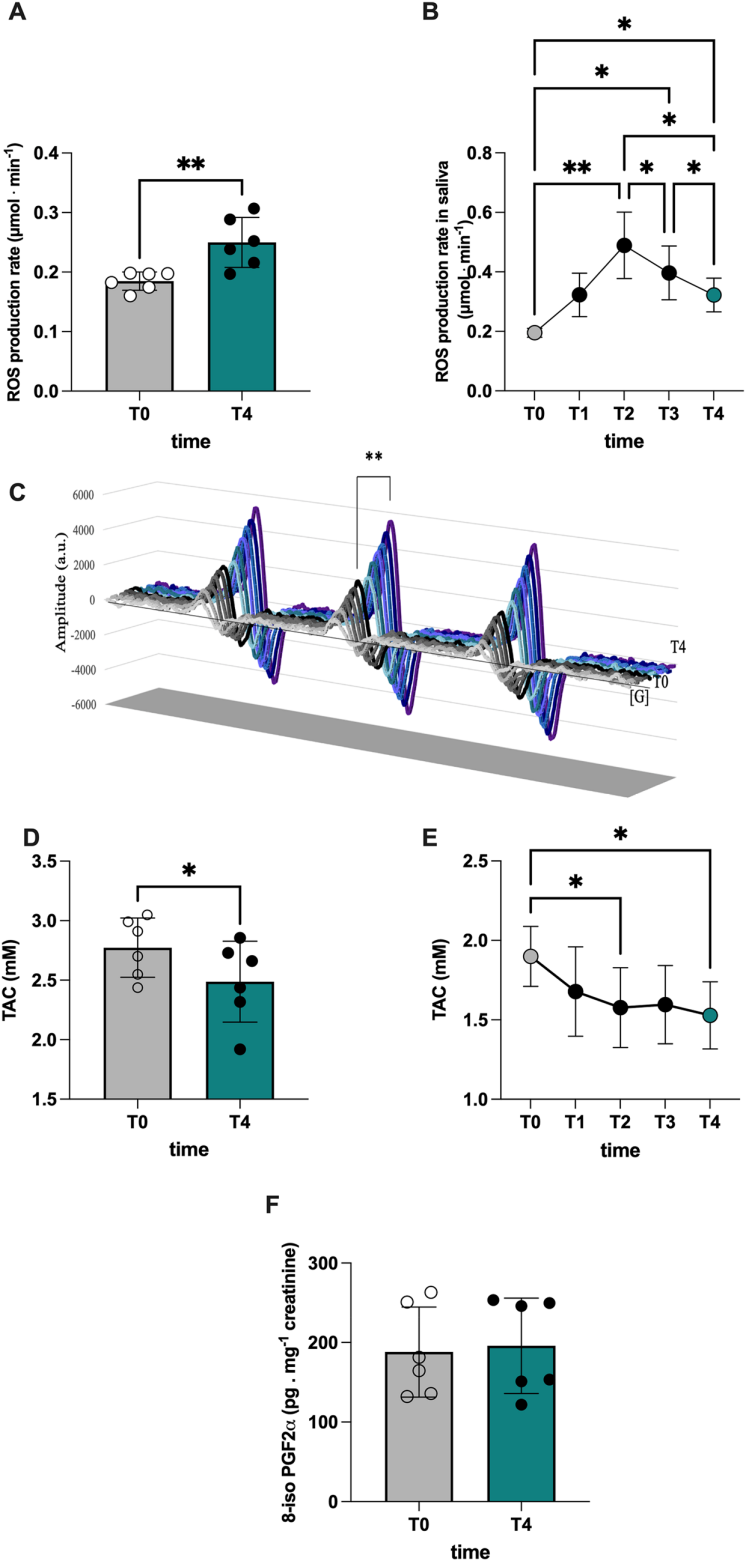
Bar chart (mean ± SD) of **A** ROS production rate. **B** Time course of ROS production rate. **C** Stacked plots of the EPR spectra show an increase of the signal amplitude (a. u.) at T4 (blu lines) with respect to T0 (black lines). The spectra are centered at *g* = 1.997. *p < 0.05 and **p < 0.0 are significantly different. **D** Bar chart of total antioxidant capacity (TAC). **E** Time course of total antioxidant capacity in saliva at T0, T1, T2, T3, and T4. **F** Bar chart of lipid peroxidation (8-isoprostane) in plasma collected at T0 and T4

Under normal physiological conditions, approximately 1.2% of inspired O_2_ is converted to reactive oxygen species (ROS), and hyperoxia increases this amount (Bruakk et al. 2014). As shown in Fig. 3A, plasma ROS emission rose substantially after the dry dive test: from 0.18 ± 0.01 (T0) to 0.25 ± 0.024 µmol.min^−1^ (T4). The saliva-based EPR approach adopted allowed to follow the kinetics of ROS production (Fig. 3B). Representative pre- and post-test EPR spectra are shown in Fig. 3C. With this tool in hand, we found that, as compared to baseline values (T0: 0.19 ± 0.01 µmol.min^−1^), ROS production rate markedly increased (range p = 0.05–0.01) during the dry dive test, reaching a peak value at a depth of − 48 msw (T2: 0.49 ± 0.11 µmol.min^−1^; *p* < 0.01) while declining at − 25 msw (T3: 0.39 ± 0.09 µmol.min^−1^). Yet, this burst remained significantly above T0 value values when reaching T4 (0.32 ± 0.06 µmol.min^−1^).

Antioxidant defenses are up-regulated in response to ROS production, and recent studies have shown that endogenous antioxidant activity can be enhanced after saturation diving (Bosco et al. 2010; Morabito et al, 2011). Our data show that total antioxidant activity (TAC) declined at T4 as compared to T0, both in plasma (− 11%; Fig. 3D) and saliva samples (− 19%; Fig. 3E). Moreover, following the TAC kinetics, we found that salivary TAC reached a nadir at T2 (1.57 ± 0.25 mM) and T4 (1.52 ± 0.21 mM) with respect to the basal level (T0: 1.91 ± 0.19 mM). Together, these data indicate that a dry test is sufficient to trigger ROS emission in highly trained breath-hold divers that are not immediately countered by a parallel rise in total antioxidant capacity. No significant differences in pre- and post-8-isoPGF2-α concentration (Fig. 3F) were found.

### ROS production correlates with bioavailable dopamine decline

**Fig. 4:**
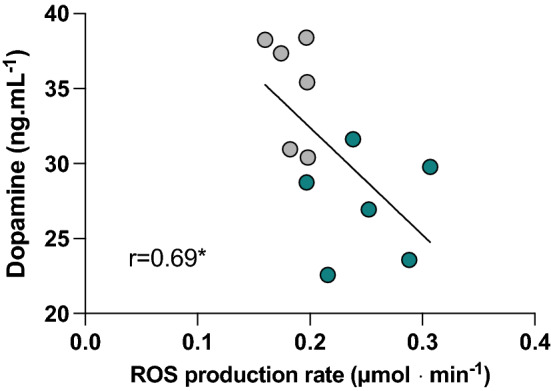
Panel plot of the relationship between ROS production rate and dopamine recorded at pre- (T0, gray circles) and post- (T4, green circles) session. The linear regression line (solid line) and the correlation coefficient (*r*) with statistical significance (*p < 0.05) is reported

We determined whether a correlation exists between the magnitude of ROS emission and dopamine levels after a dry dive test. As shown in Fig. 4A, by considering T0 and T4 times taken all together versus a single specific biomarker, a significant (*p* < 0.05) linear inverse relationship appears to exist between ROS emission and dopamine levels (*r* = 0.69; and ϕ = 3.31).

### A dry test in the hyperbaric chamber fuels pro-oxidizing and pro-inflammatory conditions in deep divers

As shown above, a dry test can trigger pro-oxidizing conditions in healthy breath-hold divers. Hence, we next interrogated whether the levels of GSH, a primary antioxidant, can underpin, at least in part, the elevated ROS emission found in these divers. Of note, there are no human data in this regard. We found that the GSH levels were significantly (*p* = 0.05) increased at T4 (Table 1), along with that of homocysteine. Conversely, the concentration of other total aminothiols, such as cysteine and cysteinyl glycine, was not significantly altered in the erythrocytes at post-deep.

**Table 1:** Redox status in erythrocytes

	Aminothiols		
	T0	T4	*p*
Cys tot ± SD	52.28 ± 9.87	46.60 ± 8.55	0.062
CysGly tot ± SD	2.434 ± 0.663	2.828 ± 0.624	0.687
Hcy tot ± SD	3.80 ± 0.77	5.60 ± 1.31	0.031*
GSH tot ± SD	2353.80 ± 90.17	2524.31 ± 93.41	0.031*

Mean (± SD) total aminothiols in RBC, values at T0 and T4. The concentrations of the various forms are expressed as μmol.L^−1^. Cys cysteine, CysGly cysteinyl glycine, Hcy homocysteine, GSH glutathione. *p* value across samples are reported.*Significantly different (*p* < 0.05)

**Fig. 5:**
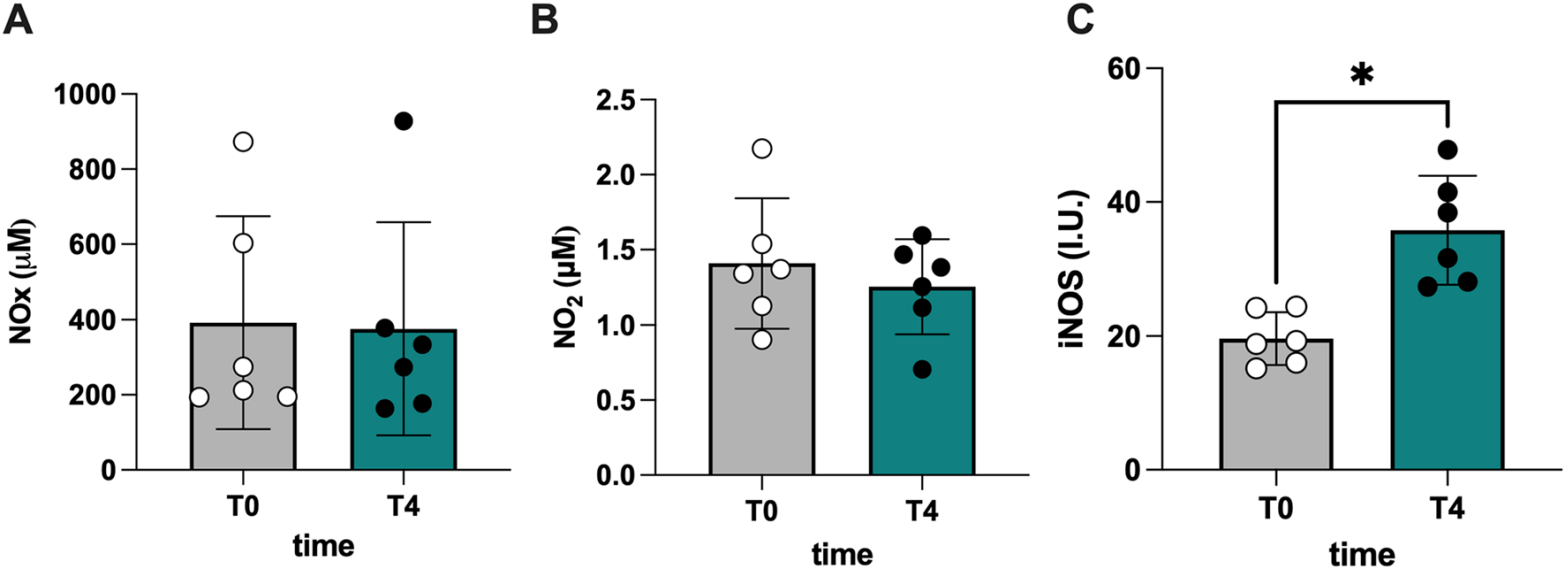
Bar charts (mean ± SD) of **A** NO metabolites (NOx), **B** nitrite (NO_2_), and **C** inducible nitric oxide synthase (iNOS), collected at T0 and T4 *p < 0.05, significantly different

Next, we tested whether a dry test (in the hyperbaric chamber) would alter the levels of NO^.^ metabolites in these divers. We observed no differences in urine between T0 and T4 in total oxidized products of NO^.^ (NOx) 391.8 ± 282.7 μM (T0) vs. 375.6 ± 283.2 μM (T4) (Fig. 5A) or nitrite (NO_2_) 1.41 ± 0.43 (T0) vs. 1.25 ± 0.31 (T4) μM (Fig. 5B), with a significant (*p* < 0.05) rise in iNOS levels, from 19.65 ± 3.95 (T0) vs 35.79 ± 8.15 IU^**.**^ mL^−1^ (T4) (Fig. 5C).

#### Neopterin and creatinine levels are elevated after a dry dive test

**Fig. 6:**
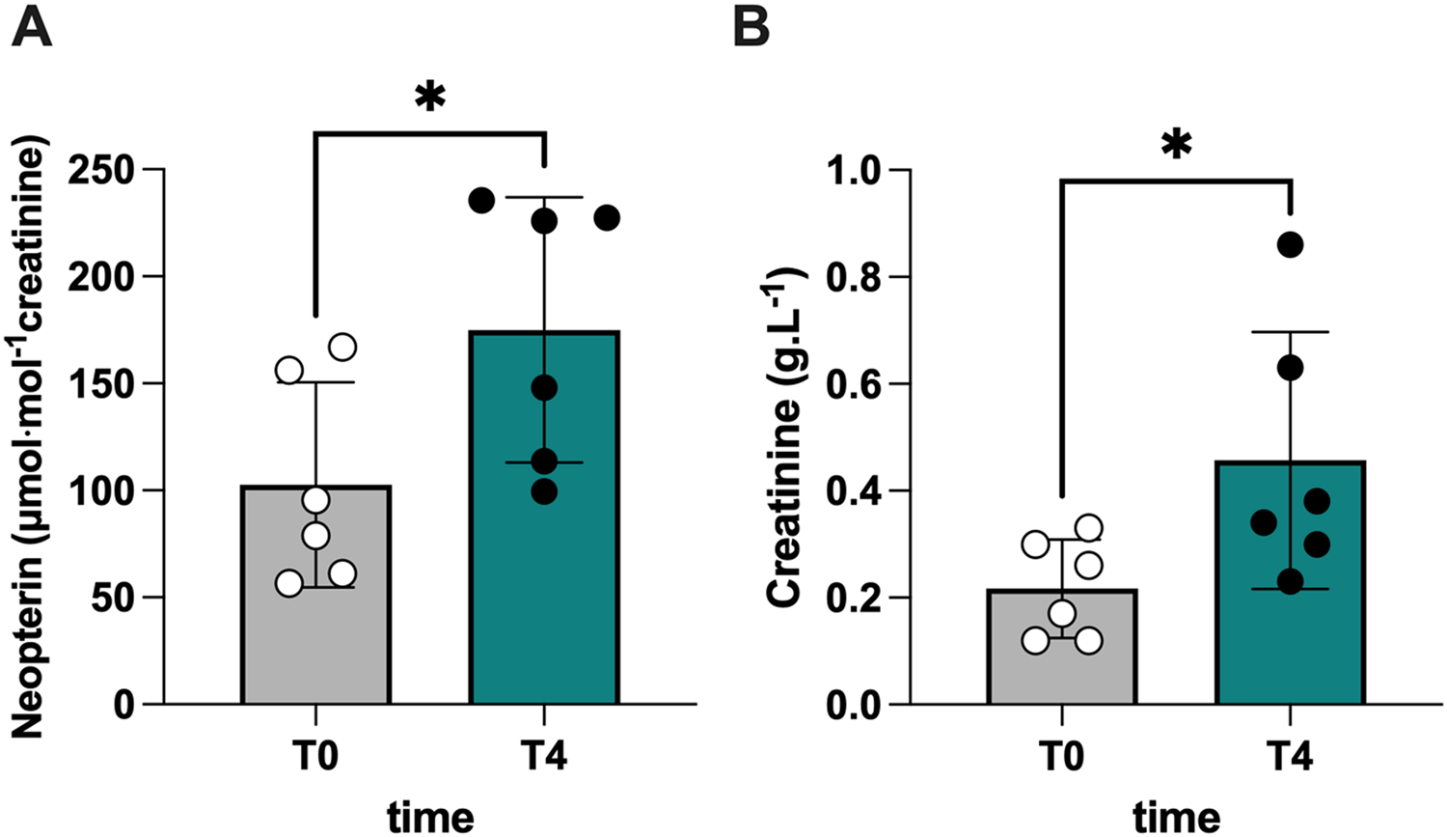
Bar charts (mean ± SD) of **A** neopterin and **B** creatinine concentration, collected at T0 and T4. *p < 0.05, significantly different

Here, we report, for the first time, that neopterin levels are more elevated at T4 than at T0: 175.0 ± 61.9 vs. 102.6 ± 47.8 (μmol.mol^−1^creatinine) (Fig. 6A), with an increase in creatinine level at T4 and T0: 0.45 ± 0.24 vs. 0.21 ± 0.09 g.L^−1^ (Fig. 6B).

## Discussion

**Fig. 7:**
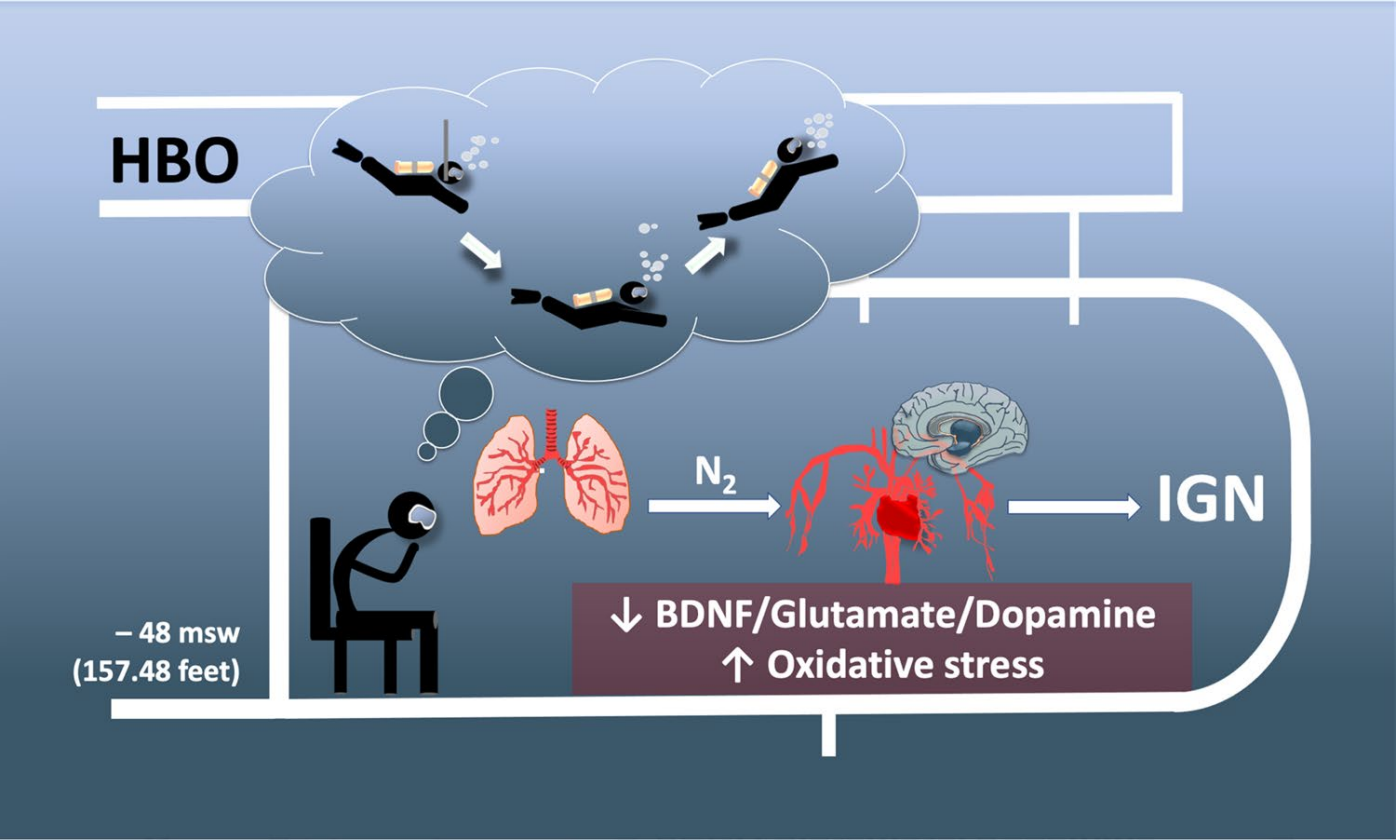
Scheme of deep narcosis test via a dry dive test (DDT) at 48 m in a multiplace hyperbaric chamber. The Inert Gas Narcosis (IGN) consists in a reversible depression of neuronal excitatory signaling - BDNF (−47%), Glutamate (−35%), Dopamine (−23%) -, and an imbalance between ROS and antioxidant defenses (i.e., ROS: +43% and TAC −10%), caused in particular by nitrogen and oxygen breathing at higher partial pressures. The influence on clinical presentation can include decreased cognitive performance. The onset varies, but can be seen around 30 msw or deeper

Hyperbaric oxygen can have toxic effects on the central nervous system (CNS) (Gasier et al. 2017), but the mechanisms whereby narcosis induces cognitive impairment in divers are unclear. The present observational study reveals, for the first time, that, after a dry dive test at depth in a hyperbaric chamber to mimic in-depth sea diving, highly trained divers manifest: (1) cognitive impairment coupled to low circulating and/or saliva content of dopamine, glutamate, and BDNF; (2) overall increased ROS emission paralleled by depleted antioxidant levels; (3) pro-oxidizing and pro-inflammatory conditions likely subtending altered vascular reactivity; and (4) elevated neopterin and homocysteine levels that may account, on the one hand, for the onset of inflammation and loss of BDNF signaling, and thus cognitive deficits, on the other (Fig. 7).

### Narcosis: short-term and long-term consequences

Narcosis-like symptoms could be common and similar between scuba and free divers; for instance, in the latter, it may occur when breath-hold divers exceed ~ 70–90 msw, leading to transient amnesia (Patrician et al. 2021a). Recently, Patrician and colleagues reported that two breath-hold divers spent 26 and 42 s, respectively, beyond 90 msw—with maximum depths of 102 and 117 msw, P_A_N_2_ increases with depth, and the total time exposed at these extreme depths appears to play a crucial role in both divers reporting narcosis (Patrician et al. 2021b). Well-trained divers adopt a range of physiological adaptations to overcome the limits; however, these modifications could interfere with some safety mechanisms that grant homeostasis and integrity of the organism (Elia et al. 2021). Long-term consequences of breath-hold diving encompass neurocognitive disorders caused by chronic exposition to hypoxia and subsequent neuroinflammation, aside from other diving-related illnesses such as blood gas embolisms (Elia et al. 2021). For example, memory impairment is not rare in these subjects compared to healthy, age-matched subjects (Billaut et al. 2018). We administered the labyrinth test or Porteus maze test (Miyake et al. 2000; Predovan et al. 2021) in hyperbaric conditions to fill this gap. Thus, the present findings extend recent evidence obtained in SCUBA divers subjected to hyperbaric chamber-simulated depth showing IGN-induced cognitive impairment during a dry dive test (Karakaya et al. 2021), which may threaten the safety of diving. Thus, as articulated above, the extent of achieved bottom depth and the time spent there are primary determinants of the partial pressure of alveolar nitrogen (P_A_N_2_), and its inordinate elevation facilitates nitrogen diffusion into the blood (Patrician et al. 2021a).

Especially when recurrent, exposures to narcosis can desensitize GABAA receptors on dopamine cells and decrease glutamate release while increasing the sensitivity of NMDA receptors, resulting in overall neurological toxic effects and motor impairment (Rostain et al. 2016). Of relevance, deficits in cognitive performance in the underwater environment are significantly more frequent in anxious subjects (Hos et al. 2011). Notwithstanding, the reasons accounting for all the phenomena described above remain ill defined. This gap in our knowledge, the consequent lack of potential interventions directed to lower IGN incidence, and the eventual severity of its remote functional and psychological repercussions deserve more investigation. On the one hand, reaching this goal will allow divers to be more focused and efficient in completing the immersion. On the other, increased knowledge and awareness, along with possible chemical or pharmacological interventions directed to attenuate the effects of narcosis will reduce the extent of an athlete's anxiety while performing deep diving and the long-term consequences of recurrent narcosis episodes.

#### Nitrogen and neurotransmitters during narcosis

According to Henry's law, nitrogen carried from the lungs dissolves in the brain, causing a drunken sensation beyond about 30 m depth. Nitrogen concentrations alter neuronal or synaptic transmission, causing impaired cognitive function, euphoria, slowing of reflexes, and stupor (Fitz-Clarke 2018). Glutamate is the major excitatory neurotransmitter in the brain (Petroff 2002). Our present data show that glutamate release is significantly decreased at T4 (− 35%). Glutamate stimulates the production of BDNF, which, in turn, modifies neuronal glutamate sensitivity, Ca^2+^ homeostasis, and plasticity (Mattson 2008). Besides trophic actions, BDNF affects synaptic transmission and plasticity, enhancing excitatory synaptic transmission through pre- and postsynaptic mechanisms, partially via augmented glutamate release (Martin et al. 2011). Vallee and colleagues (2009) have shown that nitrogen reduces the extent of glutamate and other amino acids in the striatum of rats compressed up to 0.1 Mpa at a rate of 0.01 Mpa/min and then up to 3 Mpa at a rate of 0.1 Mpa/min in a hyperbaric chamber, accounting for IGN-motor and cognitive disturbances in nitrogen narcosis (Valee et al. 2009). Yet, whether circulating BDNF levels are altered in IGN-experiencing subjects remains to be tested. Moreover, lack of dopamine and glutamate stimulation can account, at least in part, for low levels of systemic BDNF in divers at the depth. This, in turn, may contribute to altered synaptic transmission, and thus cognitive impairment in divers at the depth, in agreement with that previously found both experimentally and in humans (Yang et al. 2002; Rostain et al. 2011). In our study, we found that a correlation exists between BDNF and glutamate levels, and this evidence goes hand in hand with the fact that the excitatory neurotransmitter glutamate and BDNF are chiefly involved in the phenomena of cellular and synaptic plasticity (Gulyaeva 2017). More specifically, our present findings help explain recent evidence showing that repeated dives, even when compliant with the most up-to-date decompression tables, can progressively lead to micro-lesions in the myelin sheet, thus accounting for altered result's neuronal function (Coco et al. 2019). In the same vein, current data concerning dopamine serve two mechanistic purposes. First, they lend support to the concept that dopamine could influence BDNF levels. Dopamine enhances BDNF protein levels, and the mRNA gene transcripts for its specific receptor, tropomyosin receptor kinase B (TrkB), in neuronal and astroglia cultures of the mouse striatum (Kuppers et al. 2001); indeed, stimulating these neurons with dopamine or a dopamine D1 receptor agonist elevates their BDNF content, suggesting that dopamine effects on GABAergic cells can intersect with BDNF action (Kuppers et al. 2001). Moreover, the overall deficit in BDNF levels can lead to an imbalance in excitatory/inhibitory neurotransmission, including dysregulation of GABAergic transmission, ultimately leading to neurodegeneration (Kim et al. 2017). Second, constitutive low BDNF levels selectively alter serotonergic neurotransmission in the hippocampus via a reduced expression, and thus the function of the serotonin reuptake transporter, SERT, leading to an hyperserotoninergic phenotype (Guiard et al. 2008). There is evidence that antidepressants, such as selective serotonin uptake inhibitors (SSRIs), can increase the incidence of narcosis (Querido 2017).

### Nitrogen and hyperoxia during narcosis

Hyperbaric hyperoxia with challenging variations in PO_2_ can develop inflammation and display augmented ROS production in mammals (Balestra et al. 2021; Bosco et al. 2018a, 2021; Morabito et al, 2011), leading to cell damage via oxidative modifications in proteins, lipids, and nucleic acids. Recently, we assessed ROS production in saliva samples by EPR spectroscopy to measure a 30-day saturation dive (Mrakic-Sposta et al. 2020). EPR is the only currently available technique capable of directly detecting oxygen-free radicals by specific spin probes (Dikalov et al. 2018). There are no studies investigating IGN-induced ROS during a dry dive test in the hyperbaric chamber using this very specific and quantitative methodology. This approach may help address a vexing question such as if the body of breath-hold or scuba divers actually emits more ROS (Mrakic-Sposta et al. 2019, 2020), what role it may have in IGN unfolding. Indeed, excessive ROS emission can impair synaptic plasticity and memory function (reviewed in Massad et al. 2011); on the other hand, ROS such as superoxide anion can directly quench NO^.^, reducing its bioavailability, and thus the biological effects (Paolocci et al. 2001). Here, we show that a dry dive test boosts ROS emission and depletes total antioxidant capacity in otherwise healthy breath-hold divers, likely preparing the ground not only for a pro-oxidizing environment, but also for a pro-inflammatory terrain, as indicated by the rise in iNOS and homocysteine levels.

Of note, in our study the athletes breathed compressed air during descent and oxygen from 12 msw during decompression. When considering the narcosis mechanisms, it should be remarked that, in general, they are very similar to those of anesthetic gases, i.e., oxygen can dissolve in the fatty substances in the neuronal membranes and, because of its *physical* effect on altering ionic conductance through the membranes, it may reduce neuronal excitability. Surprisingly, however, a few studies made a vis-à-vis comparison between breathing air and enriched air nitrox (EAN) on IGN, and with controversial outcomes. Some highlighted worse psychomotor performance when using O2, EANx, or TRIMIX (Lafere et al. 2016, 2019; Balestra et al. 2018; Rocco et al. 2019). Conversely, others concluded that narcotic impairment was the same, although divers may perceive otherwise using TRIMIX (Piispanen et al. 2021).

It is worth stressing that we chose to let them breathe oxygen during decompression to obtain a nitrogen washout in our volunteers engaged in the diving training program the day after the experiment. Thus, throughout the test, the divers may have breathed a relevant hyperoxic gas mixture, almost 1.2 bar O_2_ at depth, and up to 2.2 bar pO_2_ for a longer time during decompression. Thus, in addition to IGN, hyperoxia may have also contributed to the symptomatology of the divers. Since high pO_2_ can cause a number of well-known effects, including sympatholytic effects (e.g., hyperoxic bradycardia) and oxidative stress, some of the effects seen at depth may relate not only to IGN, i.e., high pN_2_, but also to high pO_2_. Moreover, hyperoxia could have an impact on all the measured parameters, in particular on ROS and TAC levels. However, the contribution of hyperoxia to IGN—both at depth and surface remains to be understood better, especially with respect to the decline in cognitive tests. This is fertile terrain for future investigation.

### Narcosis: antioxidant and immune activation during inflammation

To further investigate possible antioxidant or pro-oxidant pathways that may be altered during the dry test in healthy breath-hold drivers, accounting for unremitted ROS emission, we first assessed the levels of glutathione (GSH), a primary antioxidant. Its role in HBO therapy remains, indeed, controversial. On the one hand, GSH appears to be up-regulated after HBO during liver regeneration in rats (Ozden et al. 2004). On the other, when investigating possible HBO-imparted protection in mice with myocardial infarction (MI), Oliveira and colleagues concluded that augmented levels of reduced GSH accentuate the pro-oxidizing conditions found in the post-MI rat heart (Oliverira et al. 2020). Of note, no data are available on GSH bioavailability at depth in humans. These alterations, in addition to the likelihood of reduced NO^.^ bioavailability, could pave the way, among other effects, to an altered arterial reactivity. In aggregate, these data corroborate our contention that a dry test in a hyperbaric chamber may lead to a pro-oxidizing environment, as attested by homocysteine and GSH level rise, and likely to pro-inflammatory conditions, as hinted at by the rise in iNOS levels. In tandem, these pro-oxidizing/pro-inflammatory conditions could acutely impair vascular reactivity. Indeed, among other effects, elevated homocysteine levels could alter arterial reactivity to adrenergic stimulation, modifying vascular sensitivity to norepinephrine, thus prompting vasorelaxation (Cipolla et al. 2000). A complex range of mechanisms conjures up in regulating the cerebral blood flow during dives. When altered, some of them could have a detrimental impact on brain functioning and can endure even after the dive (Caldwell et al. 2020; McKnight et al. 2021). Accordingly, it is well consolidated that even a single air dive can cause transient endothelial dysfunction (Culic et al. 2014), and breath-hold diving can induce significant alterations in blood gases and partial pressures in relatively short periods and shallow depths (Bosco et al. 2018b-c; 2020). Among other possible causes, all of this is not foreign to altered circulating BDNF levels. Indeed, as recalled above, in patients with psychiatric disorders, markedly reduced BDNF levels correlate with low superoxide dismutase and glutathione peroxidase, with a substantial rise in malondialdehyde (MDA) and end product of lipid peroxidation (Zhang et al. 2015). On the one hand, the activation of the dopamine D1-like receptor induces the expression of BDNF in cortical, striatal, and hippocampal rat tissue slices (Williams et al. 2009). On the other, dopamine oxidative catabolism by monoamine oxidases (MAO) B leads to an ROS outburst (Kaludercic et al. 2014a, b). Against this background, we sought to determine whether a correlation exists between the extent of ROS production and dopamine levels after a dry dive test. Hence, our data suggest that low dopamine levels could account for lower circulating BDNF levels, and thus cognitive deficit, while hinting at the intriguing possibility that its accelerated catabolism by MAO may contribute to the overall rise in ROS emission seen in deep divers.

Neopterin is a clinical marker of immune activation during inflammation, and it has been widely studied, particularly under stressful conditions (Gieseg et al. 2018). Moreover, high levels of this potent antioxidant could be generated by interferon-γ-activated macrophages and derive from the oxidation of 7,8-dihydroneopterin. Of note, neopterin levels are elevated in brain illnesses, such as depression (Celik et al. 2010; Maes et al. 2012). Intriguingly, when subjected to repetitive transcranial magnetic stimulation (rTMS), patients suffering from depression manifest symptom improvement, along with an inverse correlation between BDNF and neopterin. In fact, after rTMS, BDNF levels are more elevated and neopterin is lower than in sham-treated patients (Leblhuber et al. 2019). This evidence further supports the notion that a cross talk exists between BDNF and neopterin bioavailability that is central to IGN pathogenesis. At this time, we can only speculate that monitoring neopterin levels can serve as a footprint of narcosis duration/intensity.

## Limitations and studies in perspective

The present study comes with some limitations that need further, in-depth investigation. First, for safety reasons, we enrolled a relatively small sample size of subjects in the hyperbaric chamber (protocol). Future studies would be conducted in larger populations at greater depths and in open water. Second, we acknowledge the observational nature of our experimental approach that, at the moment, precludes answering some salient mechanistic questions, such as factors leading to the decline of the systemic levels of dopamine, glutamate, and BDNF. We anticipate this question can be satisfactorily addressed only using animal models where sources of ROS can also be dissected in great detail. In the same vein, studying IGN onset and unfolding in small rodents would help determine if and in which brain regions pools of BDNF and key neurotransmitters, such as dopamine and glutamate, are more severely affected by single or repeated episodes of IGN, and same for the status of the BDNF-specific receptors, TrkB. All these relevant questions warrant future in-depth investigation and a more nuanced assessment of vascular reactivity.

## Conclusion

Our study shows that healthy divers challenged with a dry dive test in a hyperbaric chamber develop narcosis and loss of cognitive function when at deep depth, i.e., 48 msw. A marked loss in circulating levels of dopamine/glutamate and BDNF at T4, i.e., 10 min after resurfacing, accompanies this phenomenon, along with altered systemic redox conditions, as revealed by the overall ROS emission detected both in the saliva and blood of these subjects, and a drop in total antioxidant capacity was shown. The present findings suggest that boosting dopamine/BDNF signaling pharmacological or behavioral interventions—pre-, during, or after diving—can attenuate narcosis and, therefore, its short- and long-term adverse effects, particularly in those subjects that undergo repeated deep diving.

## Data Availability

All raw data that were presented or mentioned in the manuscript are available from the corresponding authors on request.

## Abbreviations

ATA: Absolute atmosphere
BDNF: Brain-derived neurotrophic factor
Cys: Cysteine
CysGly: Cysteinyl glycine
CNS: Central nervous systems
DA: Dopamine
DDT: Dry dive test
EPR: Electron paramagnetic resonance
GSH: Glutathione
HBO_2_: Hyperbaric oxygen
Hcy: Homocysteine
HPLC: High-performance liquid chromatography
IGN: Inert gas narcosis
iNOS: Inducible nitric oxide synthase
NE: Norepinephrine
NO: Nitric oxide
NO metabolites: NO_2_, + NO_3_ = NOx
OxS: Oxidative stress
ROS: Reactive oxygen species
TAC: Total antioxidant capacity
8-iso-PGF2α: 8-Isoprostane
